# Mechanisms of Epistemic Change—Under Which Circumstances Does Diverging Information Support Epistemic Development?

**DOI:** 10.3389/fpsyg.2018.02278

**Published:** 2018-11-22

**Authors:** Martin Kerwer, Tom Rosman

**Affiliations:** Leibniz Institute for Psychology Information (ZPID), Trier, Germany

**Keywords:** epistemic beliefs, epistemic change, psychology, diverging information, experimental study, gender stereotypes, higher education

## Abstract

**Background:** The number of studies on how to foster change toward advanced epistemic beliefs (i.e., beliefs about the nature of knowledge and knowing) is continuously growing because these beliefs are an important predictor of learning outcomes. In past intervention studies, presenting diverging information (e.g., descriptions of studies yielding contradictory results) reliably led to epistemic change. However, prior research insufficiently examined which aspects of diverging information affect these changes.

**Aims:** We investigated (1) if epistemic change differs depending on the (un)resolvability of contradictory information, (2) to what extent explicitly reflecting on diverging information supports epistemic change and (3) how topic-specific diverging information affects topic–and domain-specific epistemic beliefs. All confirmatory hypotheses were preregistered at OSF. Additionally, several exploratory analyses were conducted.

**Method:** To examine the research questions, we employed a simple randomized pre-post design with four experimental groups. *N* = 185 psychology students participated in the study. Experimental groups differed in the kind of diverging information included: Students either read (1) information on students applying learning strategies (control), (2) unresolvable, or (3a) resolvable controversial information on gender stereotyping. In the latter condition (3b), an additional group of participants deliberately resolved apparent contradictions in a writing task.

**Results:** Confirmatory latent change analyses revealed no significant group differences in epistemic change (i.e., beliefs in the control group also changed toward advanced epistemic beliefs). Using a different methodological approach, subsequent exploratory analyses nevertheless showed that presenting diverging information on gender stereotypes produced stronger topic-specific epistemic change and change in justification beliefs in the treatment groups in contrast to the control group. However, effects in the treatment groups did not differ significantly depending on the resolvability of presented controversies or for the group which was instructed explicitly to integrate controversial findings.

**Conclusion:** Contrary to our expectations, diverging information seems to foster epistemic change toward advanced beliefs regardless of the resolvability of presented information, while no final conclusion concerning effects of reflection could be drawn. Moreover, our findings indicate that effects of topic-specific interventions are more pronounced on topic-specific measures. However, this relationship may vary depending on the epistemic belief dimension (e.g., justification beliefs) under investigation.

## Introduction

Epistemic beliefs are conceptualized as an individual's beliefs about the nature of knowledge and knowing (Hofer and Pintrich, 1997). Even though a long tradition of interdisciplinary research on the predictors and effects of epistemic beliefs exists (Hofer and Pintrich, 1997; Greene et al., 2008, 2018; Chinn et al., 2011), interventions that aim to promote epistemic change are relatively rare (cf. Muis et al., 2016). Recently, however, interest in epistemic change surged (Kienhues et al., 2016; Muis et al., 2016; Barzilai and Chinn, 2017). This may, at least partially, be due to the fact that these beliefs have been repeatedly shown to affect how individuals deal with crucial requirements of a modern knowledge-based society, such as acquiring and evaluating knowledge (Kienhues et al., 2016; Strømsø and Kammerer, 2016). Accordingly, quasi-experimental and correlational studies point toward beneficial effects of advanced epistemic beliefs (e.g., beliefs that knowledge claims have to be weighed and evaluated) for information integration (Barzilai and Ka'adan, 2017) and sourcing (Bråten et al., 2014), while more naive types of beliefs tend to impair the performance in such tasks (e.g., Kammerer et al., 2015; Rosman et al., 2016b). In this context, the term *naive beliefs* embraces views that (1) knowledge claims can only be either true or false, or (2) the conception of knowledge as purely tentative and subjective (Kuhn et al., 2000). In line with these ideas, a recent meta-analysis by Greene et al. (2018) confirmed that epistemic beliefs are positively correlated with academic achievement, which further corroborates the importance of (fostering) those beliefs.

To allow for future intervention studies to shape individuals' epistemic development in a more efficient way, our research aims to contribute to a better understanding of the underlying mechanisms of change. In this article, we start by briefly introducing popular developmental models for epistemic beliefs, as well as established models on epistemic change and models on the domain-specificity of epistemic beliefs. Thereafter, we review recent approaches for changing epistemic beliefs in (quasi-) experimental settings, focusing on the presentation of diverging information as an especially promising method. Bringing together these theoretical perspectives, we identify three essential and unsettled research questions that relate to properties of diverging information and the domain-specificity of both the presented information and the beliefs under investigation. Subsequently, we introduce an experimental study that addresses these research questions by examining psychology students' epistemic beliefs on gender stereotyping in secondary schools. Finally, after presenting the study's results, we discuss its implications for both future research on epistemic change and for the design of interventions that target epistemic change.

### Developmental models on epistemic beliefs

How are changes in epistemic beliefs thought to take place in non-experimental settings throughout an individual's lifespan? Most developmental models for describing epistemic change strongly rely on Piagetian ideas introducing *cognitive disequilibrium* as the driving force behind epistemic development (Hofer and Pintrich, 1997). More specifically, these models assume that cognitive disequilibria occur if new information contradicts previously acquired beliefs. For example, belief change may occur when math students realize that there is more than one way to solve problems in mathematics. Again typically Piagetian, almost all established developmental models postulate that epistemic development unfolds in distinct stages. In this study, we draw on the popular model of Kuhn et al. (2000), who propose a stage model that differentiates three stages of epistemic beliefs: Individuals start as *absolutists*, believing that knowledge is certain and that an objective truth exists. They then proceed to *multiplism*, whose characteristic aspect is that knowledge is seen as inherently subjective. The final and most advanced stage is called *evaluativism*, where individuals acknowledge the importance of weighing evidence and integrating contradictory knowledge claims. In our opinion, this does not imply that evaluativists deny the existence of certain knowledge. For example, an evaluativist may argue strictly in favor of vaccination if there is sufficient evidence to support its efficacy. Additionally, in a modern society with divided knowledge, advanced beliefs may also involve acknowledging one's knowledge gaps, identifying trustworthy external authorities that address these gaps (e.g., the World Health Organization for health issues), and relying on the information provided by them (Bromme et al., 2010). According to Kuhn et al. (2000), individuals successively progress from absolutism over multiplism to evaluativism in their epistemic development (although not all individuals reach the last stage). On a more fine-grained level, one may additionally characterize these rather broad stages on a set of dimensions so-called integrative models (e.g., Bendixen and Rule, 2004; Merk et al., 2018) with *certainty, simplicity, justification* and *source* of knowledge being the most prominent ones (Hofer and Pintrich, 1997). However, it should be of note that Greene et al. (2008) challenge this view by arguing that some of those dimensions, such as *simplicity* of knowledge, relate to an individual's ontological beliefs and not to their epistemic beliefs. Therefore, they suggest focusing on justification beliefs as “truly” epistemic beliefs that determine under which circumstances individuals obtain knowledge. For this purpose, Greene et al. (2008) introduced two dimensions of justification beliefs – *justification by authority* (e.g., individuals justify knowledge claims based on experts) and *personal justification* (e.g., justification of knowledge claims based on personal experience). Subsequently, Ferguson et al. (2012) extended this framework by adding a third scale, *justification by multiple sources*, whose importance was confirmed by ensuing studies (e.g., Bråten et al., 2013).

### Mechanisms of epistemic change—the bendixen-rule model

Bendixen and Rule's (2004) *process model for personal epistemology development* describes more precisely how cognitive disequilibria presumably cause epistemic change in a certain situation. It introduces three central prerequisites of epistemic change (i.e., epistemic doubt, epistemic volition and resolution strategies), which are parts of a higher order mechanism (Bendixen, 2016). An idealized description of the proposed mechanism of change in Bendixen and Rule's model is as follows: As a starting point of epistemic change, an individual experiences epistemic doubt, a cognitive dissonance. This dissonance leads to questioning one's epistemic beliefs and may occur as a response to new information that contradicts an individual's existing beliefs (Rule and Bendixen, 2010). In order to deliberately tackle this epistemic doubt, it requires a certain amount of epistemic volition (i.e., the “will” or motivation for epistemic change), the second central component of the model (Rule and Bendixen, 2010). Thereafter, epistemic doubt is resolved by applying resolution strategies, such as reflection or social interaction, and individuals eventually adopt more advanced beliefs (Bendixen and Rule, 2004). However, proceeding to advanced beliefs is not guaranteed, even if all of these components are activated. Indeed, individuals may even regress to more naive beliefs under specific circumstances (Bendixen and Rule, 2004), which are, unfortunately, only vaguely specified in the original model. However, the notion that *epistemic doubt* may occur at any stage of an individual's epistemic development (i.e., even evaluativists are expected to question their beliefs from time to time) entails some important implications when designing intervention programs. To name only one, the interplay between prior beliefs and intervention contents has to be carefully considered (cf. Rule and Bendixen, 2010). Thus, the same instructional approach may be fruitful for absolutists, while it at the same time unintentionally evokes doubt on evaluativists' advanced beliefs. Nonetheless, this model is not uncontested, and, as Bråten (2016) stressed, the empirical validation of many assumptions of Bendixen's model, including its proposed mechanism of change, is still largely unsatisfactory.

### Domain-specificity of epistemic beliefs and epistemic change

So far, we treated epistemic beliefs in a universal way, thereby implying that beliefs on knowledge and knowing do not differ depending on the content domain they relate to. Indeed, epistemic development was initially considered to be consistent across fields or domains, and earlier research (e.g., Schommer, 1993) almost exclusively used this domain-general approach (i.e., it was assumed that individuals possess similar epistemic beliefs across content domains). Recent research has challenged this assumption by showing that epistemic beliefs encompass both domain-specific and domain-general aspects that are shared across domains (Buehl and Alexander, 2005; Muis et al., 2006). Moreover, Bråten and Strømsø (2010) argue that the same principle may also apply to specific topics, such as gender stereotyping, within certain domains or subdomains, for instance educational psychology. They further argue that the impact of epistemic beliefs on educational outcomes (such as academic achievement) should be particularly strong if beliefs and outcomes are measured on the same level of specificity. Drawing upon this thought, intervention-induced epistemic change should be particularly strong in epistemic belief measures whose specificity corresponds to the specificity of the information used to evoke epistemic doubt and subsequent changes in epistemic beliefs. Even though this assumption may sound highly plausible—especially as it is in line with findings from social psychology on the role that relevant exemplars play in behavior change (e.g., Lockwood and Kunda, 1997; Han et al., 2017), its empirical backing is certainly extendable.

### Experimentally inducing epistemic change

After providing this overview of the framework in which epistemic change is thought to occur, the question of how to efficiently influence individuals' epistemic development remains. As the number of research programs dedicated to achieve this aim is constantly growing, a variety of intervention approaches has been developed (see Bendixen, 2016; Muis et al., 2016). Naturally, it is theoretically sound and intuitive to evoke enduring belief change in long-term intervention programs, for example by using constructivist teaching methods (e.g., Muis and Duffy, 2013). However, short-term experimental interventions have recently become more prominent (Kienhues et al., 2016). A major advantage of this study type is that it allows for a better control of experimental circumstances and for a more specific investigation of the psychological mechanisms involved in epistemic change (even though far from all short-term interventions make use of this advantage). Moreover, those interventions have been shown to be surprisingly effective in inducing epistemic change—at least in the short term (Kienhues et al., 2008, 2011; Ferguson and Bråten, 2013). Most prominently, the presentation of *diverging information* (i.e., information that includes contradictory knowledge claims) has been shown to reliably evoke epistemic change (Kienhues et al., 2016), indicating that cognitive disequilibria (and subsequent epistemic doubt) are likely to be a driving force of epistemic development. Several interventions have been designed on this basis (Kienhues et al., 2016). For example, Kienhues et al. (2011) confronted students with conflicting knowledge claims concerning medication use for the control of cholesterol and showed that topic-specific epistemic change was more pronounced under these circumstances when compared to students that received consistent information on this topic.

Regrettably, however, most of these intervention studies fail to specify the kind of change in epistemic beliefs that is desired (Bråten, 2016); such as if they intend to reduce naive beliefs or foster advanced beliefs. Especially studies that are not strongly based on Kuhn's framework often seem to strive to simply reduce absolute beliefs and tend to neglect possible adverse effects of strong multiplistic beliefs. More precisely, frequently proposed adverse effects of multiplism encompass impaired viewpoint and text comprehension (Bråten et al., 2013; Barzilai and Eshet-Alkalai, 2015) as well as impeded sourcing (Barzilai et al., 2015). Thus, even though the mere presentation of conflicting (or diverging) information has been shown to efficiently reduce absolutism, such interventions do not ensure that evaluativistic beliefs prosper. In fact, it is much more likely that an individual will simply “replace” absolute beliefs with multiplistic beliefs or that already existing multiplistic views are strengthened when he or she is confronted with inconsistent evidence on a specific topic. Furthermore, from a theoretical point of view, one may suggest that backward transitions from evaluativism to multiplism might occur if individuals are repeatedly confronted with diverging information including controversies that are more difficult to integrate (e.g., the conflicting intervention condition of Kienhues et al., 2011). As outlined above, this kind of epistemic change is, in our view, not worth striving for. Therefore, we need interventions that make individuals avert both absolute and multiplistic beliefs, while at the same time supporting a change toward evaluativistic beliefs.

### The resolvable controversies intervention

To address this need, Rosman et al. (2016a) developed an intervention approach, which—by drawing on so-called *resolvable controversies*—aims to reduce both absolutism *and* multiplism simultaneously, as well as to foster evaluativism. On a global level, it illustrates, based on apparently conflicting findings of studies on gender stereotyping at secondary schools, how to identify contextual factors that help to explain controversies when evidence seems to be ill-structured—or, more strictly speaking, it exemplifies how to weigh knowledge claims (Rosman et al., 2016a).

Recently, Rosman and Mayer (2018) used the following procedures for implementing the intervention: First, 18 short abstracts of conflicting studies on gender stereotyping and gender-specific discrimination in schools are presented. A crucial component of the resolvable controversies intervention is that apparent contradictions in these texts can be resolved (or integrated) by identifying the context in which a certain type of discrimination (favoring either boys or girls) occurs. To support this process, participants are additionally asked in adjunct questions who is discriminated against according to the present study. For example, intervention contents imply that girls are discriminated against in physics while boys are discriminated against in languages and literature. In this case, participants are thought to identify the factor “subject matter” as a contextual factor that explains apparent inconsistencies between the studies. This resolvability of apparent contradictions is thought to induce epistemic doubt concerning both absolutism and multiplism because a variation in findings exists but is explainable (Rosman et al., 2016a). According to Rosman, Mayer and Merk (under review), this insight should subsequently be generalized to higher-level domains (e.g., educational psychology). Unfortunately, on an empirical level, prior studies did not explicitly confirm this assumption—for example, by introducing a control condition drawing on inexplicable discrepancies in findings (i.e., “unresolvable” controversies)—but focused on the overall efficacy of the intervention instead.

In the second part of Rosman and Mayer's (2018) intervention, subjects proceeded by integrating conflicting findings in a writing task. In the resolution instruction of this writing task (i.e., the most prolific instruction for eliciting epistemic change), subjects were required to complete a scientific essay which illustrates conditions of gender-specific discrimination based on the presented studies. Because of the didactical properties of the presented controversies, subjects are expected to identify the aforementioned contextual factors under these circumstances. As the effects of both parts of the intervention (i.e., the reading and writing tasks) have never been disentangled, it remains unclear to what extent the intervention's efficacy can be attributed to either one of both of those distinct intervention contents. Examining these reading and writing tasks separately would be particularly insightful for clarifying how deeply diverging information has to be processed in order to affect epistemic beliefs. For example, drawing upon Bendixen and Rule's model of epistemic change, the writing task might trigger the resolution of epistemic doubt that was evoked by the presentation of diverging information. The underlying mechanism would be that a reflection on conflicting information in presented texts (during the writing task) prompts a reflection on one's own epistemic doubt that has been evoked by the respective texts. Although some studies investigated links between explicit reflection on epistemic beliefs and subsequent changes in those beliefs (see Lunn Brownlee et al., 2016), prior research failed to address the distinct relationship between receiving diverging information, reflecting on it, and epistemic change.

### Research questions

Based on these considerations, the purpose of our study is to shed some light onto how exactly diverging information may foster change toward advanced epistemic beliefs. Our first research question aims at identifying specific circumstances and characteristics of diverging information that trigger change toward certain types of epistemic beliefs.

1) Under which circumstances does diverging information evoke epistemic change toward advanced belief types (i.e., no simple reduction of absolutism at the cost of rising multiplistic beliefs, but a reduction of both absolutism and multiplism, and a simultaneous change toward evaluativism)?

Moreover, we want to examine the effects of a deep processing of diverging information by separating effects of the *presentation* of diverging information (which should be closely related to the occurrence of epistemic doubt) from effects of *reflecting* on this information (which is possibly connected to the resolution of this doubt). Thus, our second research question is:

2) Will interventions based on resolvable controversies still be able to induce epistemic change toward advanced epistemic beliefs after removing all components that are linked to reflecting on how to integrate conflicting information?

As described above, it is plausible to assume that changes in epistemic beliefs depend on the level of specificity of both the administered intervention (i.e., presented diverging information) and the epistemic belief measure used. More specifically, intervention effects may be stronger if both levels of specificity correspond to each other. In our last research question, we will empirically scrutinize this assumption and examine to what extent changes in topic-specific beliefs (e.g., beliefs regarding the topic of gender stereotypes) carry over to higher-level domains (e.g., beliefs regarding educational psychology).

3) Are the effects of topic-specific epistemic change interventions more pronounced in topic-specific epistemic belief measures?

In the next section, materials and methods of our study designed specifically to answer these questions are described.

## Materials and methods

All planned procedures and hypotheses of our confirmatory analyses have been preregistered at the Open Science Framework (https://osf.io/te7wk/). For the reader's convenience, they are re-iterated here. Moreover, this section also includes information on actually collected data, exploratory outcomes and exploratory analyses. All study measures and methods were in compliance with the Declaration of Helsinki and the APA Ethics Code (American Psychological Association, 2002). Ethical approval was obtained from the Ethics Committee of the German Psychological Association and prior to their participation, all students gave their informed consent. Since study inclusion and pre-intervention measurements were conducted online, no written informed consent could be obtained at study inclusion. However, we provided an information sheet and consent form (for download) and subjects were only allowed to enter the study if they confirmed (by checking a box) that they agreed to the conditions specified in these documents. As all other study measures, these procedures for online data collection and study inclusion were approved by the Ethics Committee of the German Psychological Association.

### Participants and study timeline

Our research questions were investigated with data from an experimental study employing a 4 × 2 pre-post design with one between-subjects factor (intervention type with four levels) and one within-subjects factor (repeated measurement factor with two levels). In total, *N* = 201 psychology students (minor and major), who were recruited at Trier University by means of flyers and mailing lists, partook in the online pre-intervention measurement. At least 1 week after this measurement, the second measurement occasion took place in group sessions at a university lab. In the second measurement occasion—that included the intervention as well as the post-intervention measurement—*N* = 185 students participated (92.04% of participants who had enrolled at the first measurement occasion) and received 20 Euro upon study completion. For one participant, pre-intervention and post-intervention data could not be matched and, thus, data of the first measurement occasion had to be treated as missing data. Thus, our dataset contains *N* = 184 students whose demographical data is known. These participants (89.67% females) had a mean age of *M* = 23.21 (*SD* = 3.13). 95.65% of our participants studied psychology as their major subject (59.78% Bachelor and 35.87% Master students), while 4.35% took a minor in psychology. The median study duration was six semesters (*M* = 5.85, *SD* = 2.97).

### Procedures and materials

#### Intervention

We modified Rosman and Mayer's (2018) resolvable controversies intervention that has been described above to address our research questions. We pursued two aims with this modification: (1) to inspect how the resolvable nature of presented controversies affects epistemic change, and (2) to examine the distinct effects of presenting diverging information (i.e., evoking epistemic doubt) on epistemic change by separating effects of doubt from effects that are possibly related to deeper level processing (i.e., the resolution strategy *reflection*).

**Figure 1:**
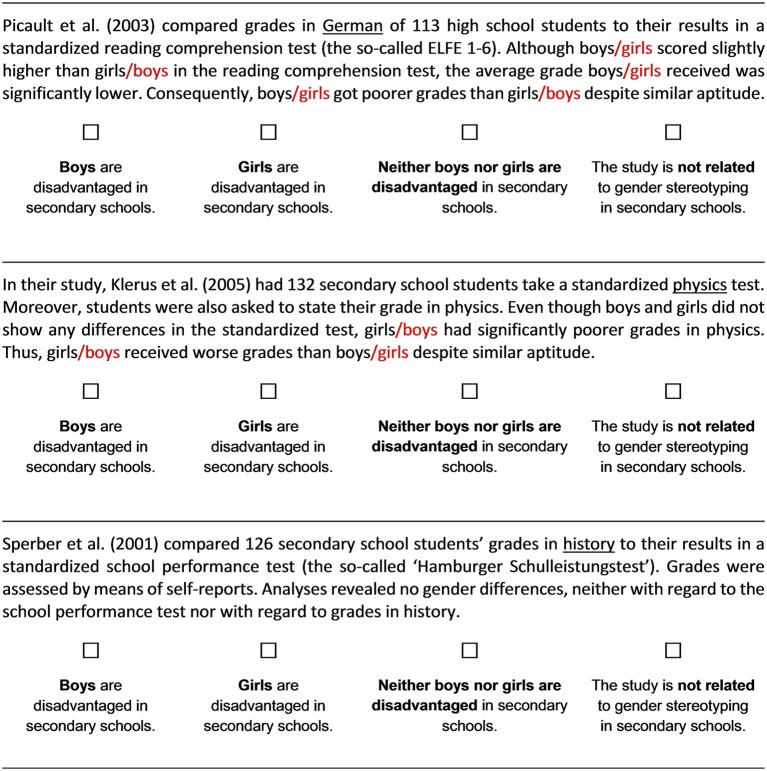
Three resolvable controversies sample texts. Cues allowing to resolve the controversies are underlined and red marks illustrate how texts were modified in the “Unresolvable Read” group (please note that only half of the cues were changed resulting in an overall random pattern of discrimination). The complete German version of the texts is available on request.

To clarify if epistemic advancement does indeed depend on the resolvability of the controversies, we “masked” the resolvable nature of these controversies by distorting the effects of contextual factors that explain diverging findings (see Figure 1 for an illustrative example). For example, if the original intervention text states that boys are consistently discriminated against in languages and literature, the modified version stated that some studies find that boys are discriminated against in languages and literature while others find that girls are disadvantaged in these subjects. Thus, we eliminated the pattern that underlies the presented conflicting information and, hence, the intervention should induce doubt concerning absolutism only because diverging findings cannot be integrated anymore. Multiplism, in contrast, might even be fostered since the abundance of conflicting information is likely to convey views of the knowledge body in question as extremely tentative and inconsistent.

Considering the second aim, that is singling out effects of epistemic doubt, we shortened the original resolvable controversies intervention of Rosman and Mayer (2018). The original paradigm uses both reading and writing about resolvable controversies. By means of specific writing instructions, participants are invited to integrate conflicting information and, thus, reflect on this information. It cannot be finally ruled out that this higher level processing of diverging information also causes reflection on participants' epistemic doubt. Thus, we separated effects of inducing epistemic doubt by the mere presentation of diverging information from effects of reflecting on this information by comparing a shortened version of the intervention, where the writing task is left out, to the original intervention that includes this writing task.

In order to test the overall efficacy of our intervention, we compared changes in epistemic beliefs in these three treatment conditions^1^ to changes in a control group. Participants in the control group read texts on students employing learning strategies. To design this task as similar as possible to the gender stereotypes reading task—which required participants to rate for each presented study if boys or girls were discriminated against (adjunct questions)—each text snippet of the control task contained two descriptions of students employing different learning strategies that were compared to each other. For example, participants learned that two students applied different approaches concerning the length and distribution of their learning units. While one student learned from 9 a.m. to 6 p.m. and only took a short lunch break of 20 min, the other student only learned for 2 h at a time and took extensive breaks in between. After reading both descriptions, participants were asked to assess the characteristics of these learning strategies on a set of scales, such as required effort or generation of detailed knowledge.

To sum up, intervention conditions or “experimental groups” in our study differed in the kind of intervention that participants received:

- *Control (learning strategies)*. Group 1 read texts on students employing different learning strategies,
- *Unresolvable Read*. Group 2 read conflicting materials which cannot be resolved by identifying moderator variables (i.e., a modified version of the conflicting materials that are used in groups 3a and 3b),
- *Resolvable Read*. Group 3a read conflicting materials whose contradictions could be resolved (i.e., the original reading task of the resolvable controversies intervention),
- *Resolvable Read and Write*. Group 3b read conflicting materials whose contradictions could be resolved (the same task that group 3a received) and was additionally subjected to the resolution writing task of the resolvable controversies intervention.

The following time limits applied to respective tasks: Participants were allowed a maximum of 15 min for the reading task and 45 min for the writing task (in group 3b).

#### Assignment to groups

Upon the start of the second measurement occasion, randomized assignment of participants to experimental groups was carried out using the respective function of the survey software Unipark. The study was single-blind (i.e., study staff could become aware of the assigned experimental group during the intervention). However, since all instructions that differed between groups and that were related to experimental manipulations were given in computerized form, this could not affect data quality. As expected, experimental groups did not differ significantly (all *p* > 0.10) in any demographic variables we assessed (i.e., age, gender, study semester, study subject, secondary school grades), nor in any pre-test scores on our dependent variables.

#### Manipulation check

To evaluate whether our manipulation worked as intended, we checked if presented information on gender stereotypes were perceived as more controversial and contradictory in the “Unresolvable Read” group when compared to the “Resolvable Read” and “Resolvable Read and Write” groups. The underlying rationale is that—since we intended to thwart the integration of conflicting results by our modification of Rosman's intervention—higher scores on perceived contradictoriness indicate that diverging information has been recognized as non-resolvable in this group.

**Figure 2:**
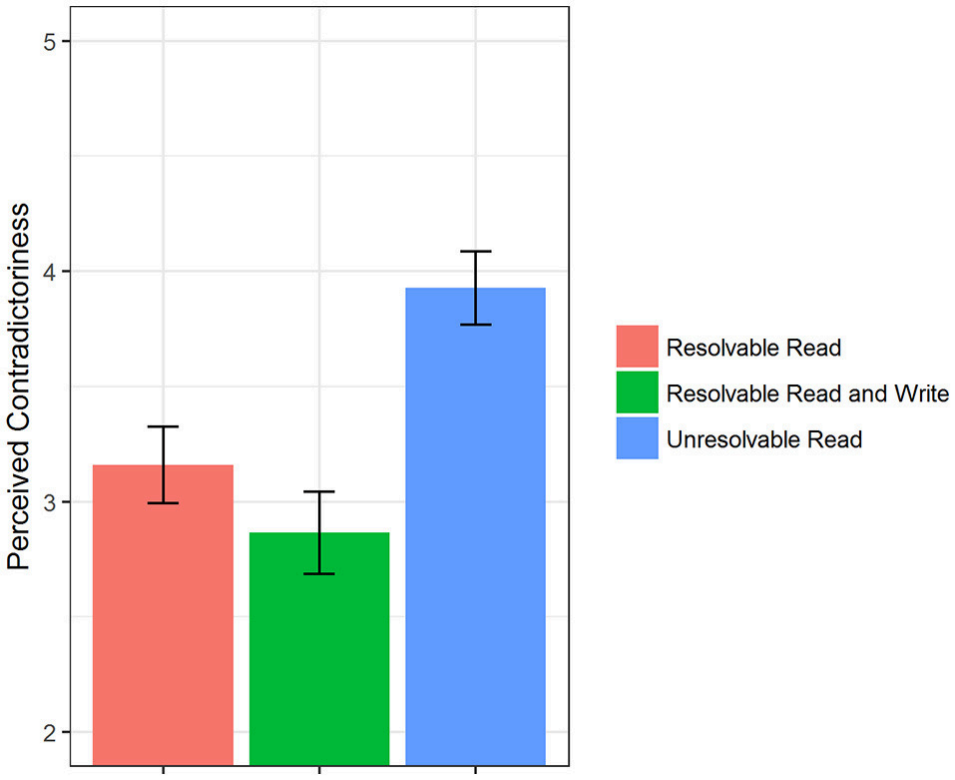
Manipulation Check. Descriptive differences (means and standard errors) in perceived contradictoriness between groups that received controversial information on gender stereotyping.

In order to test whether the expected differences occurred, we employed a self-report questionnaire that assessed to what extent subjects perceived presented information on gender stereotyping to be controversial or conflicting. A sample item is “Upon reading the texts…findings seemed to be very contradictory.” The reliability on this scale was good (Omega total ranging from 0.80 to 0.81 in the three treatment groups). As a statistical technique, we used multiple regression analyses with the “Unresolvable Read” group as reference category and dummy-coded variables for group membership as predictors. It should be of note that the contradictoriness was only assessed for the “Unresolvable Read,” “Resolvable Read” and “Resolvable Read and Write” group because of its topic-specific focus. Assessment took place after the intervention was finished in respective groups (i.e., after reading the controversies in the “Unresolvable Read” and “Resolvable Read” group and after writing a text on these controversies in the “Resolvable Read and Write” group). Figure 2 provides a graphical overview of reported contradictoriness' mean scores separated by intervention group.

Results of these multiple regression analyses revealed that the perceived overall contradictoriness of presented information differed significantly between groups, *R*^2^ = 0.13, *F*_(2, 136)_ = 10.61, *p* < 0.001. More precisely, estimates for dummy-coded regression coefficients indicate that subjects in the “Unresolvable Read” group rated presented information to be more inconsistent than subjects in both the “Resolvable Read” group (*b* = −0.77, *t*_(136)_ = −3.213, *p* < 0.01) and the “Resolvable Read and Write” group (*b* = −1.06, *t*_(136)_ = −4.468, *p* < 0.001).

Thus, our manipulation succeeded in “masking” the resolvability of inconsistent findings which is an integral part of the original intervention. Participants in the “Unresolvable Read group judged information concerning gender stereotypes to be more controversial than subjects in the “Resolvable Read” and “Resolvable Read and Write” groups.

#### Dependent variables

Confirmatory dependent measures are the FREE-GST, a topic-specific measure of epistemic beliefs and the FREE-EDPSY, a domain-specific measure of epistemic beliefs. Both measures are based on Kuhn et al. (2000) framework and were initially developed and validated in a recent study of Rosman, Mayer and Merk (under review).

##### Primary outcome: topic-specific epistemic beliefs (FREE-GST)

The FREE-GST measures topic-specific epistemic beliefs on gender-stereotype discrimination in secondary schools. The questionnaire starts with the presentation of three controversial positions on gender stereotype discrimination (i.e., boys are disadvantaged, girls are disadvantaged, neither boys nor girls are disadvantaged). Thereafter, 15 statements on this controversy, which represent either absolute, multiplistic, or evaluativistic beliefs, are to be rated on a 6-point Likert scale (5 statements per belief type). A sample item for evaluativism is “Gender specific discrimination can be diverse. Accordingly, depending on certain contextual factors, rather one or the other view is correct.”

##### Secondary outcome: domain-specific epistemic beliefs (FREE-EDPSY)

The FREE-EDPSY applies the same procedure to domain-specific epistemic beliefs in educational psychology. It introduces controversial scientific positions relating to the domain of educational psychology (i.e., an argument about the efficacy of an unspecified method of this field, such as a learning strategy or a teaching method). Subsequently, just like in the FREE-GST, 15 statements relating to either absolute, multiplistic, or evaluativistic beliefs are presented. A sample item for multiplism is “In educational research, scientists interpret their findings based on their personal opinion. Actually, nobody can know for sure whether specific methods are beneficial for learning or not.”

##### Computation of scales and indices for the FREE-GST and FREE-EDPSY

Absolutism, multiplism and evaluativism scores were computed as mean scores of the respective items for the FREE-GST and FREE-EDPSY, exactly as has been done in prior research (e.g., Rosman and Mayer, 2018). After inspecting psychometric properties of these scales, we decided to drop one item of the multiplism scale because reliabilities increased for both the FREE-GST and the FREE-EDPSY if this item was excluded.

Furthermore, we combined absolutism, multiplism and evaluativism scores to the so-called D-index, which Krettenauer (2005) proposed as an overall measure of advanced epistemic beliefs. Applying Krettenauer's formula to our questionnaires, the D-index was computed as *Evaluativism –.5 x (Absolutism* + *Multiplism)* for the FREE-GST and the FREE-EDPSY. Because the D-Index condenses changes across absolutism, multiplism and evaluativism, we expected the power to detect such overall changes toward advanced beliefs to be higher in analyses using the D-Index. However, as the D-index was not part of our preregistration, analyses including this index are exploratory.

##### Exploratory outcome: psychology-specific justification beliefs

We assessed psychology-specific justification beliefs by a domain-specific adaptation of a domain-general German questionnaire (Klopp and Stark, 2016). Klopp and Stark's questionnaire builds on items originally developed by Ferguson et al. (e.g., Bråten et al., 2013; Ferguson and Bråten, 2013). The questionnaire differentiates the three types of justification beliefs that were introduced above: (1) personal justification, (2) justification by authority, (3) justification by multiple sources. All scores were computed as mean scores.

#### Covariates

To control for influences of third variables, we measured a set of potential covariates. Need for cognitive closure was assessed by Schlink and Walther's (2007) questionnaire as connections to epistemic change have already been empirically shown for this construct (Rosman et al., 2016a). Additionally, (Bendixen and Rule, 2004) repeatedly emphasized the (theoretical) importance of environmental factors. In order to account for this, we employed Schiefele and Jacob-Ebbinghaus (2006) study satisfaction questionnaire. Moreover, as Bendixen and Rule's model on epistemic change is closely connected to conceptual change theory (Bendixen and Rule, 2004), covariates that are proposed in the conceptual change literature, i.e. need for cognition, task value, prior topic interest and self-reported prior knowledge (Dole and Sinatra, 1998; Sinatra and Mason, 2013), were included as well. Therefore, we employed an established measurement instrument by Bless et al. (1994) for need for cognition and a questionnaire that proved to reliably assess task value dimensions in prior research (Gaspard et al., 2017). Since these variables were only included in exploratory analyses if they differed at least marginally significantly between groups (see below), further details are only provided for control variables that are relevant for the present paper in **Tables 2, 3**.

### Hypotheses

Based on the research questions that were introduced above, we derived the following hypotheses:

*H1*. Epistemic belief change can be induced by text-based interventions that evoke epistemic doubt. The predicted patterns of epistemic change regarding the three developmental stages of epistemic beliefs (absolutism, multiplism, evaluativism) can be found in Table 1.

**Table 1:** Predicted pattern of effects (FREE-GST, FREE-EDPSY).

	**Absolutism**	**Multiplism**	**Evaluativism**
Resolvable read and write	–	–	++
Resolvable read	–	–	+
Unresolvable read	–	+	0
Control (learning strategies)	0	0	0

*+, increase in epistemic beliefs; –, decrease in epistemic beliefs; 0, no change in epistemic beliefs*.

More specifically, we expect small to moderate effects for the following differences between intervention conditions:

- *H1a*. Reading multiple texts presenting conflicting scientific evidence will induce epistemic change, whereas reading texts on students employing different learning strategies will not induce epistemic change.
- *H1b*. Evaluativism will increase if the conflicts between the texts may be resolved by identifying moderator variables (‘resolvable controversies') compared to a condition including texts in which the conflicts cannot be resolved.
- *H1c*. The ‘resolvable controversies' intervention reliably induces epistemic change even if it is shortened by leaving out the writing task. Incremental effects of the writing task will be small to moderate.

*H2*. All effects on epistemic change will be more pronounced in the topic-specific measure FREE-GST compared to the domain-specific FREE-EDPSY questionnaire.

In the following, statistical procedures for testing these hypotheses are described.

### Statistical analyses

All statistical analyses were conducted in R 3.5.0 (R Core Team, 2018). The package lavaan 0.6-1 (Rosseel, 2012) was used for latent variable analyses.

#### Statistical model

##### Confirmatory analyses

**Figure 3:**
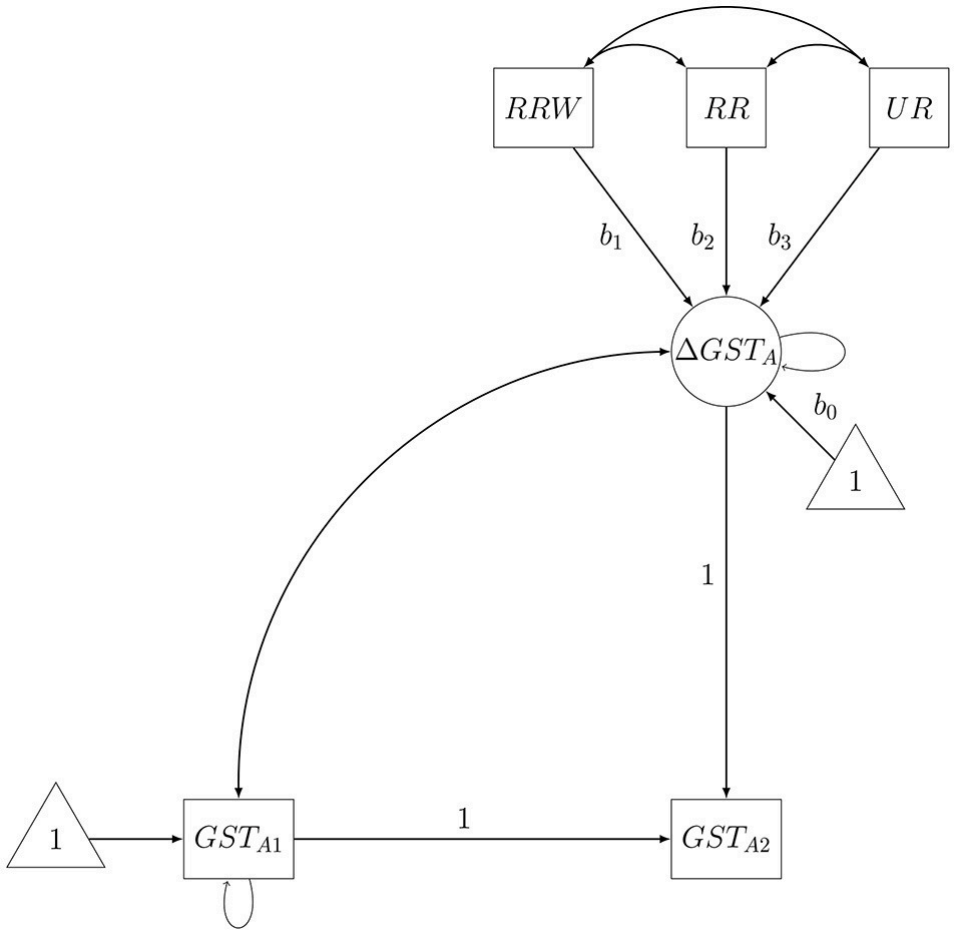
Exemplary latent change model for testing H1. Latent change in epistemic beliefs Δ *GST*_*A*_ (i.e., latent change in absolutism on the FREE-GST) is predicted by dummy-coded variables indicating group membership (i.e., *RRW* for “Resolvable Read and Write”, *RR* for “Resolvable Read” and *UR* for “Unresolvable Read”). Latent change itself is operationalized as the part of an observed outcome variable *GST*_*A*2_ (i.e., absolutism on the FREE-GST post-intervention) that differs from its pre-intervention measurement *GST*_*A*1_ (i.e., absolutism on the FREE-GST pre-intervention).

We used latent difference score modeling (McArdle, 2009) to analyze our data. The main outcome variables of our analyses were changes in epistemic beliefs (i.e., absolutism, multiplism and evaluativism scores of the FREE-GST and FREE-EDPSY), which were operationalized as latent change scores (see Figure 3 for more details). These latent change scores were predicted by dummy-coded intervention group variables. In order to investigate group differences not related to the reference group, we defined these effects as new parameters of the structural equation model. The same procedure holds for comparisons between topic–and domain-related measures (H2). Analyses concerning H1 were conducted separately for absolutism, multiplism and evaluativism (for FREE-GST and FREE-EDPSY, respectively) resulting in a total number of six target models. A logical precondition of H2 (more pronounced effects on epistemic change for the topic-specific FREE-GST) is that group differences in epistemic change exist. Therefore, H2 was only to be tested if any significant group differences were found in analyses that are related to H1. However, H2-analyses were performed even if the revealed pattern of effects contradicted the hypothesized pattern of effects. H2-analyses were conducted separately for absolutism, multiplism and evaluativism resulting in a maximum possible number of three target models.

The following procedure was employed for testing our hypotheses: First, intervention group was dummy-coded with the control group as reference category^2^. Thereafter, we estimated a null model that fixed differences in epistemic change between groups (*b*_1_ = *b*_2_ = *b*_3_ = 0) [H1] or between topic-specific and domain-specific measures (*b*_0*GST*_ = *b*_0*EDPSY*_, *b*_1*GST*_ = *b*_1*EDPSY*_, *b*_2*GST*_ = *b*_2*EDPSY*_*, b*_3*GST*_ = *b*_3*EDPSY*_) [H2] to zero. Subsequently, we compared this null model to a target model that imposed no restrictions on differences in epistemic change between groups (*b*_1_ = *x*_1_*, b*_2_ = *x*_2_, *b*_3_ = *x*_3_) [H1] or topic–and domain-specific measures (*b*_0*GST*_ = *x*_4_, *b*_0*EDPSY*_ = *x*_5_, *b*_1*GST*_ = *x*_6_*, b*_1*EDPSY*_ = *x*_7_, *b*_2*GST*_ = *x*_8_, *b*_2*EDPSY*_ = *x*_9_*, b*_3*GST*_ = *x*_10_, *b*_3*EDPSY*_ = *x*_11_) [H2]. If the corresponding likelihood ratio test (LRT) revealed that epistemic change differed significantly between groups [H1] or measures [H2], we inspected the estimated model parameters in order to examine group [H1] or measure differences [H2] in epistemic change. We used the standard *p* < 0.05 criteria for likelihood ratio tests and for determining if the estimated effects of (dummy-coded) intervention group variables were significantly different from those expected if the null hypothesis was correct. As the expected direction of effects as well as the expected order of effects is explicitly predicted, we used one-tailed tests whenever appropriate.

##### Exploratory analyses

In addition to this preregistered procedure, we introduced an alternating model which proposed that the presentation of topic-specific diverging information had an overall effect on epistemic beliefs that was invariant across treatment groups (i.e., in the “Resolvable Read,” “Resolvable Read and Write” and the “Unresolvable Read” group). Strictly speaking, this “equal group effects” model thereby suggests that neither the writing task nor the resolvable or unresolvable nature of the intervention materials mattered, but that the mere presentation of diverging information may trigger epistemic change. In order to specify this model, we restricted effects of dummy-coded variables to be equal across treatment conditions (*b*_1_ = *b*_2_ = *b*_3_) and repeated our analyses for the FREE-GST and FREE-EDPSY. Furthermore, we analyzed the five additional exploratory outcomes introduced above: justification beliefs (personal justification, justification by authority, justification by multiple sources), and the D-Indices of the FREE-GST respectively the FREE-EDPSY.

As a consequence, we extended our model comparison procedure for choosing a target model as follows: In a first step, we compared the equal group effects model (*b*_1_ = *b*_2_ = *b*_3_) to the null model (*b*_1_ = *b*_2_ = *b*_3_ = 0) based on a likelihood ratio test. The selected model of the first step was subsequently compared to our target model from the confirmatory analyses (*b*_1_ = *x*_1_*, b*_2_ = *x*_2_, *b*_3_ = *x*_3_). Otherwise, we applied the same procedures as for confirmatory hypothesis testing.

We also checked for pre-test differences on covariates that were measured before group assignment took place by means of ANOVAs with group as factor. If any marginally significant or significant differences between groups on covariates existed, we conducted additional analyses that introduced these covariates as predictors of both pre-intervention beliefs and epistemic change in our latent change model.

Finally, we investigated if the intervention was especially beneficial for subjects that held more naive epistemic beliefs (i.e., prior beliefs as indicated by pre-intervention values). For this purpose, we divided our sample into groups with more naive or more advanced epistemic beliefs—as has been done in prior research on epistemic change (e.g., Kienhues et al., 2008). More precisely, we repeated all prior exploratory analyses that yielded significant intervention effects and used multiple group modeling to test if these intervention effects differed between naive and advanced groups. For each multiple group model, we split our sample into a naive and an advanced group based on the median score of pre-intervention values of the outcome variable under investigation and tested if intervention effects differed between these groups based on LRTs.

#### Statistical power and sample size calculation

Our a priori determined target sample size was 212 participants (i.e., 53 for each experimental group). In order to calculate this target sample size, we conducted a simulation study in R. For each condition of this simulation study (i.e., tested sample size), we generated 1,000 datasets and, subsequently, analyzed the data using the statistical model described above. The expected effect size in the population model of this simulation study was derived from a previous study by Rosman, Mayer and Merk (under review), who examined epistemic change using the resolvable controversies intervention and employed a similar design to our current study. In this study, the authors showed that modifying the resolvable controversies intervention by introducing alternating writing tasks caused significant differences in epistemic change between conditions (i.e., a standardized regression coefficient of 0.276 for change in evaluativism). As we assumed that dropping the writing task or changing the resolvable nature of the presented controversies were much stronger modifications of the established resolvable controversies intervention, we expected larger effects in the current study. Our simulation study revealed that such effects would be detectable for a sample size of *n* = 53 subjects per group: The power for detecting small to moderate effects (i.e., beta = 0.40), which range above the practical significance criterion introduced by Ferguson (2009), surpassed 85%. Moreover, the power for detecting moderate effects (i.e., beta = 0.50) was above 96% for this sample size. A reanalysis with our actual sample size (46 subjects per group) showed that the power for detecting small to moderate effects still approximated 80% and was therefore acceptable.

## Results

Reliabilities and intercorrelations of all study variables for the first measurement occasion are given in Table 2, while means and standard deviations (separated by group) are given in Table 3. Moreover, considerable ceiling effects existed for the *justification by multiple sources* scale (pre 15.22% and post 20.00% of all subjects showed values at the upper limit of the scale), as well as small ceiling effects for *evaluativism* on both the FREE-GST (2.72% pre and 8.11% post) and the FREE-EDPSY (6.52% pre and 7.57% post). Floor effects for all other measures were neglectable (< 5.00% pre respectively 6.50% post), while the D-Index was completely unaffected by ceiling effects. There were no univariate or multivariate outliers on dependent variables according to the criteria of our preregistration (i.e., based on *z*-scores with *p*(*z*) < 0.001 for univariate outliers and a mahalanobis distance with *p*(χ^2^, *df* = 6) < 0.001 for multivariate outliers). Thus, no outlier-corrected analyses were performed.

**Table 2:** Intercorrelations and reliabilities of study variables at the pre-intervention measurement occasion (t1).

	**Correlation \*p*-values**	**1**	**2**	**3**	**4**	**5**	**6**	**7**	**8**	**9**	**10**	**11**	**12**	**13**
1	Absolutism (topic-specific)	**0.73**	0.047	< 0.001	< 0.001	0.017	< 0.001	0.014	0.007	0.327	0.132	0.479	< 0.001	< 0.001
2	Multiplism (topic-specific)	0.147	**0.69**	0.004	0.764	< 0.001	0.014	< 0.001	0.605	0.707	0.088	0.338	< 0.001	< 0.001
3	Evaluativism (topic-specific)	−0.313	−0.212	**0.71**	0.002	0.007	< 0.001	< 0.001	0.845	0.019	0.002	0.056	< 0.001	< 0.001
4	Absolutism (domain-specific)	0.662	0.022	−0.224	**0.76**	0.512	0.001	0.167	< 0.001	0.554	0.390	0.922	< 0.001	< 0.001
5	Multiplism (domain-specific)	0.176	0.701	−0.199	0.049	**0.79**	0.017	< 0.001	0.184	0.504	0.793	0.710	< 0.001	< 0.001
6	Evaluativism (domain-specific)	−0.274	−0.180	0.757	−0.253	−0.176	**0.69**	0.002	0.738	0.036	0.019	0.299	< 0.001	< 0.001
7	Personal Justification	0.181	0.623	−0.262	0.102	0.711	−0.229	**0.77**	0.198	0.131	0.953	0.714	< 0.001	< 0.001
8	Justification by Authority	0.198	−0.038	−0.014	0.254	−0.098	−0.025	−0.095	**0.76**	0.056	0.364	0.505	0.336	0.324
9	Justification by Multiple Sources	−0.073	−0.028	0.172	−0.044	0.050	0.154	0.112	−0.141	**0.73**	0.604	0.404	0.043	0.201
10	Task Value	−0.112	−0.126	0.226	−0.064	−0.020	0.173	0.004	0.068	0.039	**0.90**	0.053	0.001	0.051
11	Prior Interest Gender Stereotypes	0.053	−0.071	0.141	0.007	0.028	0.077	−0.027	−0.050	0.062	0.143	**0.77**	0.188	0.641
12	D–Index (topic–specific)	−0.641	−0.562	0.845	−0.407	−0.455	0.661	−0.469	−0.071	0.150	0.236	0.098	–	< 0.001
13	D–Index (domain–specific)	−0.514	−0.430	0.661	−0.577	−0.568	0.818	−0.497	−0.073	0.095	0.145	0.035	0.783	–

*N = 184 and 183 (for correlations involving prior interest in gender stereotypes or task value); values in bold on the diagonal = Omega Total; the lower triangle contains correlation estimates while the upper triangle represents corresponding p-values (two-tailed tests)*.

**Table 3:** Means and standard deviations of all study variables separated by intervention group.

	**Pre-intervention (t1)**								**Post-intervention (t2)**							
	**M_LS_**	**SD_LS_**	**M_UR_**	**SD_UR_**	**M_RR_**	**SD_RR_**	**M_RW_**	**SD_RW_**	**M_LS_**	**SD_LS_**	**M_UR_**	**SD_UR_**	**M_RR_**	**SD_RR_**	**M_RW_**	**SD_RW_**
Absolutism (topic-specific)	2.671	0.850	2.813	0.751	2.822	0.694	2.847	0.740	2.383	0.832	2.396	0.777	2.509	0.714	2.238	0.884
Multiplism (topic-specific)	2.978	0.738	3.076	0.758	2.924	0.673	3.059	0.747	2.924	0.859	2.777	0.738	2.614	0.737	2.777	0.911
Evaluativism (topic-specific)	4.871	0.606	4.870	0.558	4.813	0.666	4.749	0.720	4.891	0.682	5.048	0.530	4.935	0.595	5.026	0.673
Absolutism (domain-specific)	2.636	0.739	2.752	0.683	2.891	0.802	2.872	0.696	2.474	0.784	2.522	0.762	2.652	0.756	2.481	0.881
Multiplism (domain-specific)	3.083	0.885	3.011	0.756	2.832	0.825	3.229	0.746	2.799	0.843	2.777	0.834	2.614	0.881	2.899	0.817
Evaluativism(domain-specific)	4.987	0.602	4.922	0.537	4.917	0.563	4.877	0.687	5.052	0.583	5.074	0.534	4.991	0.537	5.111	0.575
D-Index (topic-specific)	2.047	1.082	1.925	0.908	1.940	0.932	1.796	1.030	2.238	1.141	2.461	0.895	2.373	0.738	2.518	0.953
D-Index (domain-specific)	2.127	0.936	2.040	0.886	2.056	0.930	1.826	0.968	2.416	0.919	2.424	0.814	2.358	0.804	2.421	0.917
Personal Justification	2.607	1.035	2.630	1.012	2.420	0.765	2.901	0.914	2.341	0.868	2.457	0.898	2.326	0.899	2.660	0.936
Justification by Authority	3.785	0.838	3.543	0.946	3.543	0.884	3.418	0.844	3.761	0.746	3.370	0.856	3.580	0.859	3.156	1.056
Justification by Multiple Sources	5.022	0.796	5.080	0.689	4.935	0.848	5.043	0.680	5.043	0.729	5.203	0.638	5.203	0.573	5.262	0.637
Task Value	2.920	0.675	2.707	0.618	2.549	0.680	2.660	0.681	–	–	–	–	–	–	–	–
Prior Interest Gender Stereotypes	4.341	1.131	4.399	1.104	4.072	0.883	4.809	1.056	–	–	–	–	–	–	–	–
Perceived Contradictoriness	–	–	–	–	–	–	–	–	–	–	3.928	1.082	3.159	1.133	2.865	1.219
*N*	45^*^	45^*^	46	46	46	46	47	47	46	46	46	46	46	46	47	47

M, arithmetic mean; SD, standard deviation; indices specify the intervention group LS, Learning Strategies (Control); UR, Unresolvable Read; RR, Resolvable Read; RW, Resolvable Read and Write.

* *Due to missing values the sample size for prior interest in gender stereotypes and task value was 44*.

### Confirmatory analyses

**Figure 4:**
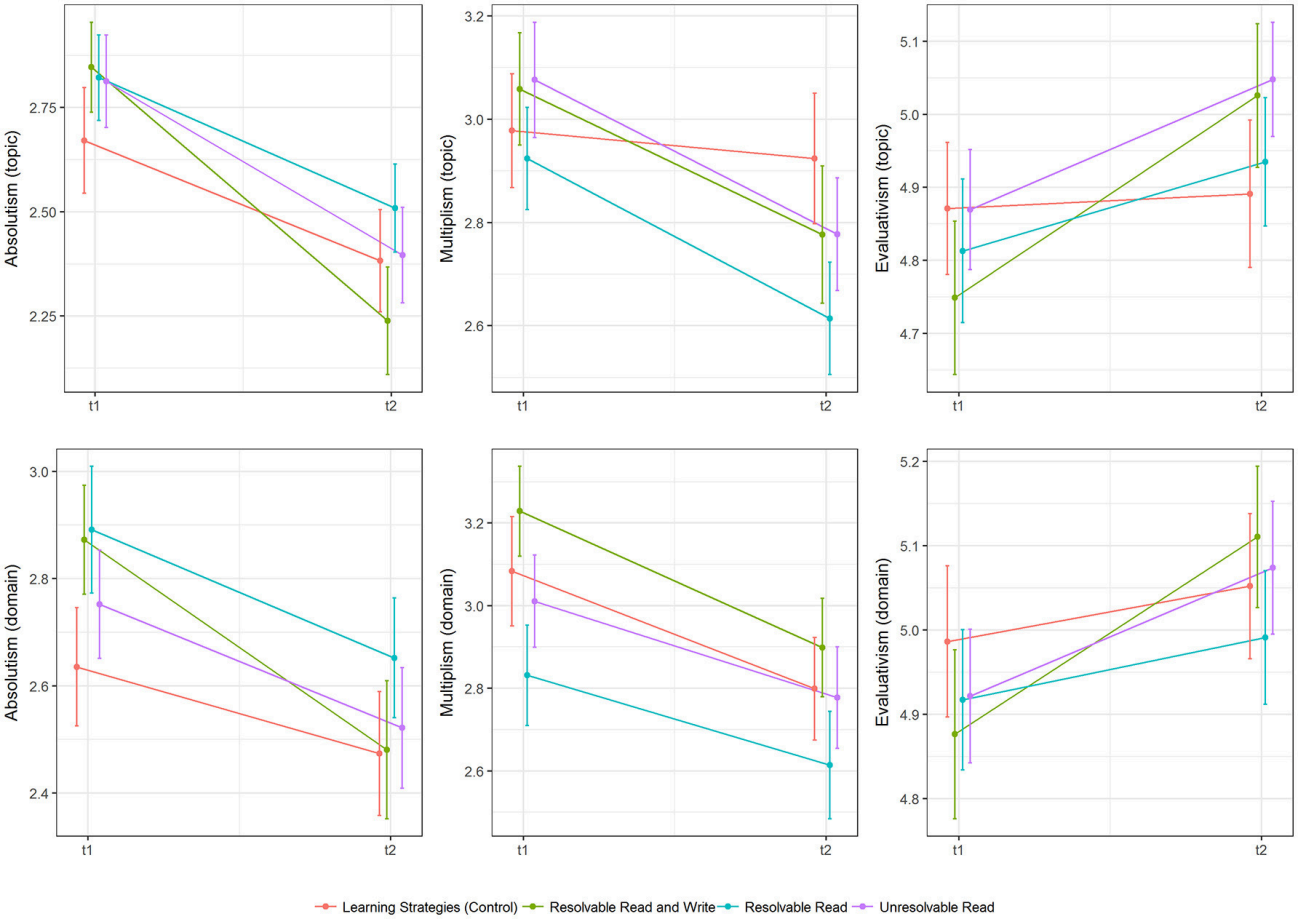
Descriptive differences (means and standard errors) in epistemic change in topic-specific (FREE-GST) and domain-specific (FREE-EDPSY) epistemic beliefs for absolutism, multiplism and evaluativism.

A graphical overview of mean changes in epistemic beliefs on primary and secondary outcomes divided by experimental groups is given in Figure 4.

#### Hypothesis 1

None of the likelihood ratio tests that were planned in our preregistration reached significance (all *p* > 0.05 see Tables 4, 5 for more details). Thus, we found no significant group differences in epistemic change according to the preregistered criterion. For topic-specific beliefs, as measured by the FREE-GST, we observed, across experimental groups, significant declines in absolutism (*b*_0_ = −0.407, *p* < 0.001) and multiplism (*b*_0_ = −0.242, *p* < 0.001), while evaluativism increased significantly (*b*_0_ = 0.153, *p* < 0.01). The same pattern was observed for domain-specific beliefs that were assessed by the FREE-EDPSY with regard to absolutism (*b*_0_ = −0.254, *p* < 0.001) and multiplism (*b*_0_ = −0.271, *p* < 0.001) and evaluativism (*b*_0_ = 0.134, *p* < 0.001, see Table 6 for more details).

**Table 4:** Fit indices and model difference tests for the FREE-GST.

	**Without covariates**							**With covariates**						
	***χ^2^***	***df***	***p***		****Δχ**^2^**	***Δdf***	***p***	**χ^2^**	***df***	***p***		**Δχ^2^**	***Δdf***	***p***
**ABSOLUTISM**														
M0. No intervention effect	**7.905**	**6**	**0.245**	M0 vs. M1	1.096	1	0.295	**7.062**	**6**	**0.315**	M0 vs. M1	1.015	1	0.314
M1. Equal group effects	6.809	5	0.235	M1 vs. M2	–	–	–	6.047	5	0.302	M1 vs. M2	–	–	–
M2: Varying intervention effects	1.673	3	0.643	M0 vs. M2	6.232	3	0.101	0.944	3	0.815	M0 vs. M2	6.118	3	0.106
**MULTIPLISM**														
M0. No intervention effect	**5.197**	**6**	**0.519**	M0 vs. M1	3.597	1	0.058	6.403	6	0.380	M0 vs. M1	3.854	1	0.0496
M1. Equal group effects	1.600	5	0.901	M1 vs. M2	–	–	–	**2.549**	**5**	**0.769**	M1 vs. M2	0.508	2	0.776
M2: Varying intervention effects	1.242	3	0.743	M0 vs. M2	3.955	3	0.266	2.041	3	0.564	M0 vs. M2	–	–	–
**EVALUATIVISM**														
M0. No intervention effect	**4.904**	**6**	**0.556**	M0 vs. M1	2.440	1	0.118	6.219	6	0.399	M0 vs. M1	4.143	1	0.042
M1. Equal group effects	2.464	5	0.782	M1 vs. M2	–	–	–	**2.076**	**5**	**0.839**	M1 vs. M2	0.455	2	0.797
M2: Varying intervention effects	1.047	3	0.790	M0 vs. M2	3.856	3	0.277	1.621	3	0.655	M0 vs. M2	–	–	–
**D-INDEX**														
M0. No intervention effect	10.502	6	0.105	M0 vs. M1	6.413	1	0.011	11.405	6	0.077	M0 vs. M1	8.079	1	0.004
M1. Equal group effects	**4.088**	**5**	**0.537**	M1 vs. M2	2.830	2	0.243	**3.326**	**5**	**0.650**	M1 vs. M2	1.800	2	0.407
M2: Varying intervention effects	1.259	3	0.739	M0 vs. M2	–	–	–	1.526	3	0.676	M0 vs. M2	–	–	–

*Boldface = target model*.

**Table 5:** Fit indices and model difference tests for the FREE-EDPSY.

	**Without covariates**							**With covariates**						
	**χ^2^**	***df***	***p***		***Δχ*^2^**	**Δ*df***	***p***	**χ^2^**	***df***	***p***		***Δχ*^2^**	**Δ*df***	***p***
**ABSOLUTISM**														
M0. No intervention effect	**6.004**	**6**	**0.423**	M0 vs. M1	0.375	1	0.540	**5.430**	**6**	**0.490**	M0 vs. M1	0.235	1	0.628
M1. Equal group effects	5.629	5	0.344	M1 vs. M2	–	–	–	5.195	5	0.393	M1 vs. M2	–	–	–
M2: Varying intervention effects	3.968	3	0.265	M0 vs. M2	2.037	3	0.565	3.457	3	0.326	M0 vs. M2	1.973	3	0.578
**MULTIPLISM**														
M0. No intervention effect	**6.141**	**6**	**0.408**	M0 vs. M1	0.033	1	0.855	**6.335**	**6**	**0.387**	M0 vs. M1	0.176	1	0.675
M1. Equal group effects	6.108	5	0.296	M1 vs. M2	–	–	–	6.159	5	0.291	M1 vs. M2	–	–	–
M2: Varying intervention effects	6.010	3	0.111	M0 vs. M2	0.131	3	0.988	6.046	3	0.109	M0 vs. M2	0.289	3	0.962
**EVALUATIVISM**														
M0. No intervention effect	**3.859**	**6**	**0.696**	M0 vs. M1	0.486	1	0.486	**3.138**	**6**	**0.791**	M0 vs. M1	1.165	1	0.280
M1. Equal group effects	3.374	5	0.643	M1 vs. M2	–	–	–	1.973	5	0.853	M1 vs. M2	–	–	–
M2: Varying intervention effects	0.704	3	0.872	M0 vs. M2	3.156	3	0.368	0.606	3	0.895	M0 vs. M2	2.532	3	0.470
**D-INDEX**														
M0. No intervention effect	**5.637**	**6**	**0.465**	M0 vs. M1	0.467	1	0.494	**4.634**	**6**	**0.592**	M0 vs. M1	0.571	1	0.450
M1. Equal group effects	5.170	5	0.396	M1 vs. M2	–	–	–	4.063	5	0.540	M1 vs. M2	–	–	–
M2: Varying intervention effects	2.640	3	0.450	M0 vs. M2	2.996	3	0.392	2.646	3	0.449	M0 vs. M2	1.988	3	0.575

*Boldface = target model*.

**Table 6:** Regression coefficients of target models predicting epistemic change in absolutism, multiplism, evaluativism and the D-Index (measured by FREE-GST and FREE-EDPSY).

	**Absolutism**				**Multiplism**				**Evaluativism**				**D-Index**			
	**No covariates**		**Covariates**		**No covariates**		**Covariates**		**No covariates**		**Covariates**		**No covariates**		**Covariates**	
	**EST**	**SE**	**EST**	**SE**	**EST**	**SE**	**EST**	**SE**	**EST**	**SE**	**EST**	**SE**	**EST**	**SE**	**EST**	**SE**
**FREE-GST**																
Intercept	−0.407^**^	0.050	−0.407^**^	0.049	−0.242^**^	0.053	–0.072	0.099	0.153^**^	0.044	0.019	0.072	0.253^*^	0.114	0.225^*^	0.113
Intervention	0.000	–	0.000	–	0.000	–	−0.226^*^	0.113	0.000	–	0.178^*^	0.085	0.300^*^	0.127	0.337^**^	0.125
Task Value		–	−0.091	0.057			0.001	0.058			0.045	0.045			0.027	0.061
Prior Interest			0.053	0.060			0.006	0.052			0.016	0.047			0.029	0.057
**FREE–EDPSY**																
Intercept	−0.254^**^	0.049	−0.254^**^	0.049	−0.271^**^	0.048	−0.271^**^	0.048	0.134^**^	0.036	0.134^**^	0.036	0.397^**^	0.054	0.397^**^	0.053
Intervention	0.000	–	0.000	–	0.000	–	0.000	–	0.000	–	0.000	–	0.000	–	0.000	–
Task Value			0.050	0.054			0.049	0.053			0.031	0.037			–0.018	0.058
Prior Interest			–0.013	0.051			–0.061	0.054			0.035	0.035			0.072	0.061
**FREE–GST and FREE-EDPSY**																
Intercept_GST_							–0.097	0.097			0.053	0.051	0.224^*^	0.107	**0.199**^+^	0.105
Intercept_EDPSY_							−0.298^**^	0.096			0.053	0.051	0.323^**^	0.091	0.334^**^	0.092
Intervention_GST_							−**0.192**^+^	0.106			**0.117**^+^	0.062	0.337^**^	0.118	0.370^**^	0.114
Intervention_EDPSY_							0.035	0.105			**0.117**^+^	0.062	0.099	0.102	0.085	0.102
Task Value_GST_							0.004	0.057			0.040	0.045			0.030	0.061
Prior Interest_GST_							0.005	0.052			0.018	0.047			0.028	0.057
Task Value_EDPSY_							0.051	0.053			0.040	0.038			0.069	0.060
Prior Interest_EDPSY_							–0.062	0.053			0.032	0.035			–0.012	0.058
Control							0.201^*^	0.093					–0.100	0.117	–0.135	0.117
Intervention							−0.227^*^	0.094					0.237^*^	0.118	0.284^*^	0.116

N = 185; reference group (0/0/0 dummy coding) = control (learning strategies); EST, unstandardized regression weight; SE, standard error; boldface scores = two-tailed significance test;

+ p < 0.10;

* p < 0.05;

** *p < 0.01*.

#### Hypothesis 2

As prespecified in our statistical analysis plan, Hypothesis 2 was not tested because confirmatory analyses concerning Hypothesis 1 revealed no significant differences between groups.

### Exploratory analyses

#### Equal group effects model

When repeating our analyses with the equal group effects model (*b*_1_ = *b*_2_ = *b*_3_), all likelihood ratio tests on primary and secondary outcomes still failed to reach statistical significance when comparing the equal group effects model to the null model (all *p* > 0.05 see Tables 4, 5 for more details).

#### D-index

**Figure 5:**
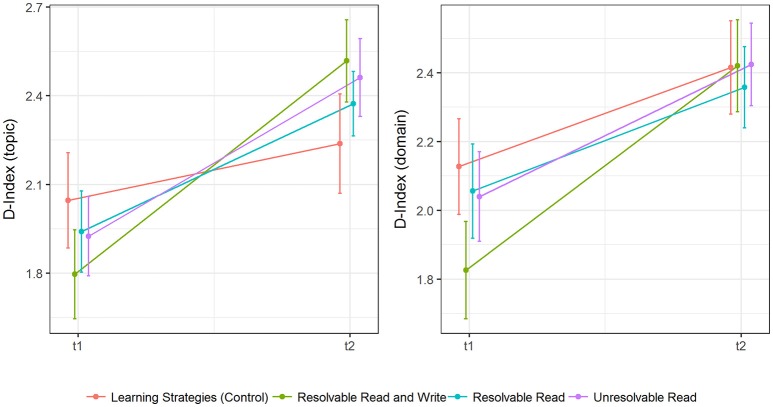
Descriptive differences (means and standard errors) in epistemic change in topic-specific (FREE-GST) and domain-specific epistemic beliefs (FREE-EDPSY) for the D-index.

Descriptive changes in the D-Index are depicted in Figure 5, while more information on descriptive statistics is available in Table 3.

For topic-specific advanced epistemic beliefs, LRTs indicated that the equal group effects model fitted our data best. In other words, effects on epistemic change for the control group and the three topic-specific intervention groups (i.e., the “Resolvable Read and Write,” “Resolvable Read,” “Unresolvable Read” groups) differed significantly (Δ χ^2^ = 6.413, *df* = 1, *p* < 0.05), while differences in effect estimates between experimental conditions did not reach statistical significance (Δ χ^2^ = 2.830, *df* = 2, *p* = 0.243). When analyzing parameter estimates of the model, we obtained the following pattern of effects: Even though D-index scores (an indicator of advanced epistemic beliefs) increased significantly in the control group (*b*_0_ = 0.253, *p* < 0.05), this increase was significantly larger across topic-specific intervention groups (*b*_1_ = 0.300, *p* < 0.05).

For the respective measure on domain-specific beliefs, LRTs indicated that neither for the equal group effects model, nor for a model with unrestricted group effects, model fit improved significantly. Across groups, we observed a significant increase in the D-Index for domain-specific beliefs (*b*_0_ = 0.397, *p* < 0.001). Tables 4, 5 provide more details on model fit difference tests and overall model fit, while Table 6 presents parameter estimates.

As epistemic change differed between groups, we tested Hypothesis 2 for the D-Index. Concerning Hypothesis 2, we selected (again based on LRTs) a model that restricted effects on topic-specific and domain-specific measures to be equal across topic-specific intervention groups (*b*_1_ = *b*_2_ = *b*_3_) but allowed these effects (and the intercept in the control group) to differ between topic- and domain-specific measures (see Table 7 for more details on model difference tests). Model inspection showed that intervention effects on epistemic change were indeed significantly more pronounced in the topic-specific D-Index than in the domain-specific D-index (*b*_1*GST*_-*b*_1*EDPSY*_ = 0.237, *p* < 0.05), while effects in the control group did not differ significantly (*b*_0*GST*_-*b*_0*EDPSY*_ = −0.100, *p* = 0.396). Again, Table 6 provides further details on parameter estimates.

**Table 7:** Fit indices and model difference tests for Hypothesis 2.

	**Without covariates**							**With covariates**						
	**χ^2^**	***df***	***p***		***Δχ*^2^**	**Δdf**	***p***	**χ^2^**	***df***	***p***		***Δχ*^2^**	**Δ*df***	***p***
**ABSOLUTISM**														
M0. No difference between measures	–	–	–		–	–	–	–	–	–		–	–	–
M1. Equal group effects for each measure	–	–	–		–	–	–	–	–	–		–	–	–
**MULTIPLISM**														
M0. No difference between measures	–	–	–		–	–	–	20.849	14	0.106	M0 vs. M1	6.329	2	0.042
M1. Equal group effects for each measure	–	–	–		–	–	–	**14.520**	**12**	**0.269**			
**EVALUATIVISM**														
M0. No difference between measures	–	–	–		–	–	–	**31.567**	**14**	**0.005**	M0 vs. M1	2.973	2	0.226
M1. Equal group effects for each measure	–	–	–		–	–	–	28.594	12	0.005			
**D-INDEX**														
M0. No difference between measures	26.781	14	0.021	M0 vs. M1	6.405	2	0.041	28.844	14	0.011	M0 vs. M1	8.550	2	0.014
M1. Equal group effects for each measure	**20.376**	**12**	**0.060**					**20.293**	**12**	**0.062**			

*Boldface = target model*.

#### Justification beliefs

**Figure 6:**
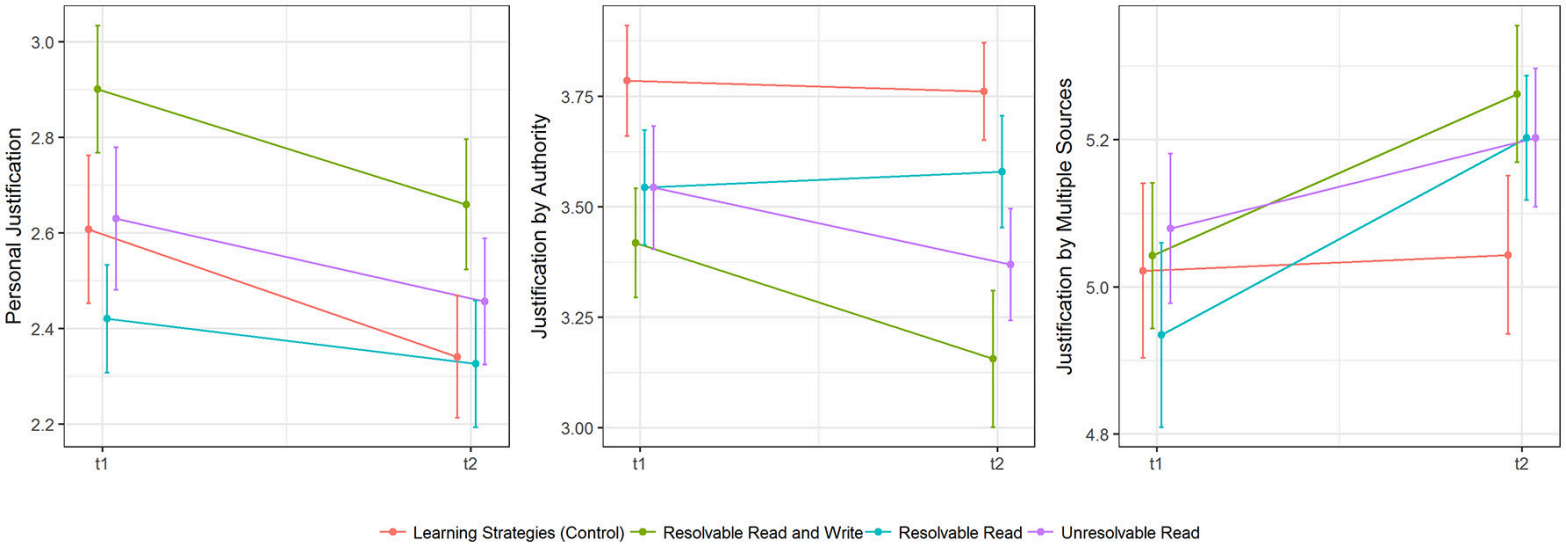
Descriptive differences (means and standard errors) in epistemic change in psychology-specific justification beliefs.

Observed changes in justification beliefs are depicted in Figure 6, while Table 8 details overall model fit and model difference tests. Finally, information on parameter estimates of the target models can be retrieved from Table 9.

**Table 8:** Fit indices and model difference tests for psychology-specific justification beliefs.

	**Without covariates**							**With covariates**						
	**χ^2^**	***df***	***p***		***Δχ*^2^**	***Δdf***	***p***	**χ^2^**	***df***	***p***		***Δχ*^2^**	***Δdf***	***p***
**PERSONAL JUSTIFICATION**														
M0. No intervention effect	**7.572**	**6**	**0.271**	M0 vs. M1	1.386	1	0.239	**8.204**	**6**	**0.224**	M0 vs. M1	1.108	1	0.293
M1. Equal group effects	6.187	5	0.288	M1 vs. M2	–	–	–	7.096	5	0.214	M1 vs. M2	–	–	–
M2: Varying intervention effects	6.154	3	0.104	M0 vs. M2	1.418	3	0.701	7.085	3	0.069	M0 vs. M2	1.119	3	0.772
**JUSTIFICATION BY AUTHORITY**														
M0. No intervention effect	13.431	6	0.037	M0 vs. M1	3.563	1	0.059	13.301	6	0.038	M0 vs. M1	2.168	1	0.141
M1. Equal group effects	9.868	5	0.079	M1 vs. M2	–	–	–	11.133	5	0.049	M1 vs. M2	–	–	–
M2: Varying intervention effects	**3.723**	**3**	**0.293**	M0 vs. M2	9.708	3	0.021	**2.940**	**3**	**0.401**	M0 vs. M2	10.361	3	0.016
**JUSTIFICATION BY MULTIPLE SOURCES**														
M0. No intervention effect	5.507	6	0.481	M0 vs. M1	4.010	1	0.045	7.050	6	0.316	M0 vs. M1	5.564	1	0.018
M1. Equal group effects	**1.497**	**5**	**0.913**	M1 vs. M2	0.557	2	0.757	**1.487**	**5**	**0.915**	M1 vs. M2	0.808	2	0.668
M2: Varying intervention effects	0.940	3	0.816	M0 vs. M2	–	–	–	0.679	3	0.878	M0 vs. M2	–	–	–

*Boldface = target model*.

**Table 9:** Regression coefficients of target models predicting epistemic change in justification beliefs.

	**Personal justification**				**Justification by authority**				**Justification by multiple sources**			
	**No covariates**		**Covariates**		**No covariates**		**Covariates**		**No covariates**		**Covariates**	
	**EST**	**SE**	**EST**	**SE**	**EST**	**SE**	**EST**	**SE**	**EST**	**SE**	**EST**	**SE**
Intercept	–0.201^**^	0.053	–0.201^**^	0.053	0.066	0.092	0.029	0.089	0.017	0.084	0.010	0.085
Resolvable read and write	0.000	–	0.000	–	–0.378^*^	0.146	–0.352^*^	0.148	0.185^*^	0.093	0.220^*^	0.095
Resolvable read	0.000	–	0.000	–	−0.037	0.128	0.044	0.123	0.185^*^	0.093	0.220^*^	0.095
Unresolvable read	0.000	–	0.000	–	–0.247^*^	0.126	–**0.211**^+^	0.119	0.185^*^	0.093	0.220^*^	0.095
Task value			−0.037	0.053			0.107^*^	0.050			0.064	0.050
Prior interest			0.035	0.050			0.066	0.051			−0.051	0.059

N = 185; reference group (0/0/0 dummy coding) = control (learning strategies); EST, unstandardized regression weight; SE, standard error; boldface scores = two-tailed significance test;

+ p < 0.10;

* p < 0.05;

** *p < 0.01*.

##### Personal justification

For *personal justification*, we found no group differences in epistemic change (*p* > 0.05 for all LRTs). Overall, personal justification beliefs decreased significantly (*b*_0_ = −0.201, *p* < 0.001) across groups.

##### Justification by authority

Regarding the next scale of the justification beliefs questionnaire, *justification by authority*, LRTs indicated that a model with varying (freely estimated) effects between experimental conditions fitted our data best (Δ χ^2^ = 9.708, *df* = 3, *p* < 0.05, see Table 8 for more details). According to this model, beliefs in justification by authority decreased significantly in the “Resolvable Read and Write” group (*b*_1_ = −0.378, *p* < 0.05) and the “Unresolvable Read” group (*b*_3_ = −0.247, *p* < 0.05) when compared to epistemic change in the control group. The corresponding effect in the “Resolvable Read” group (*b*_2_ = −0.037, *p* = 0.771) and overall change in the control group (*b*_0_ = 0.066, *p* = 0.477) did not reach statistical significance.

##### Justification by multiple sources

Finally, we selected a model with effects that were fixed to be equal for all groups that received a topic-specific intervention on gender-stereotypes for *justification by multiple sources* (Δ χ^2^ = 4.010, *df* = 1, *p* < 0.05, see Table 8 for more details). Participants of the treatment groups showed a change toward stronger beliefs in *justification by multiple sources* (*b*_1_ = 0.185, *p* < 0.05) when compared to participants in the control group whose beliefs remained unchanged (*b*_0_ = 0.017, *p* = 0.836).

#### Controlling for pre-test differences on covariates

Analyses on pre-intervention differences on covariates revealed that groups differed at least marginally significant on self-reported intrinsic task value, i.e. a positive attitude toward dealing with psychological science, *F*_(3, 179)_ = 2.47, *p* < 0.10, η^2^ = 0.04, and prior topic interest, i.e. self-reported interest in the topic gender stereotyping, *F*_(3, 179)_ = 3.93, *p* < 0.01, η^2^ = 0.06, at the first measurement occasion (and therefore prior to group assignment). More specifically, Tukey-*post-hoc*-tests indicated that participants who were later assigned to the “Resolvable Read” group had significantly lower values (*p* < 0.05) on the intrinsic task value scale when compared to the control group and on prior topic interest when compared to the “Resolvable Read and Write” group. Apart from that, no *post-hoc* comparisons yielded significant results. Due to the randomized assignment of participants to intervention conditions, these differences can only be attributed to mere chance. To deal with the issue, however, we included these variables as covariates that predicted pre-intervention differences in epistemic beliefs and epistemic change in our analyses and repeated all analyses specified above. To facilitate interpreting results of these analyses, both covariates were *z*-standardized prior to inclusion.

Results of the controlled analyses differed for topic-specific beliefs on multiplism and evaluativism. For both multiplism and evaluativism, as measured by the FREE-GST, we chose an equal group effects model (*b*_1_ = *b*_2_ = *b*_3_) based on LRTs (see Table 4 for more details). Parameter estimates of these models indicate that epistemic beliefs in the control group did not change significantly (multiplism: *b*_0_ = −0.072, evaluativism: *b*_0_ = 0.019, both *p* > 0.05). When compared to these effects, we observed a significantly more pronounced decline in multiplism (*b*_1_ = −0.226, *p* < 0.05) and increase in evaluativism (*b*_1_ = 0.178, *p* < 0.05) across topic-specific intervention groups.

Subsequently, we also tested Hypothesis 2 on multiplism and evaluativism while controlling for pre-test differences. For multiplism, an equal group effects model was chosen based on LRTs (see Table 7 for more details). Inspection of parameter estimates revealed that treatment effects were significantly more pronounced in topic-specific measures (*b*_1*GST*_–*b*_1*EDPSY*_ = −0.227, *p* < 0.05) while epistemic change toward advanced beliefs in the control group was significantly more prominent in domain-specific measures (*b*_0*GST*_–*b*_0*EDPSY*_ = 0.201, *p* < 0.05). For evaluativism, model fit did not significantly increase upon allowing effects to differ between domain-specific and topic-specific measures (see Table 7 for more details) and therefore, a model that restricted intercept and slope to be equal across topic–and domain-specific measures was chosen. Parameter estimates for this model imply that evaluativism scores in the control group did not change significantly over time (*b*_0_ = 0.053, *p* = 0.298) while in comparison a significant increase of evaluativism was detected across measures for the treatment groups (*b*_1_ = 0.117, *p* < 0.05; one-tailed). In other words, epistemic change in evaluativism does not differ between topic–and domain-specific beliefs (and H2 is therefore rejected), while an overall increase in topic–and domain-specific evaluativistic beliefs is observed for the treatment groups. Apart from these findings, results did not differ for any other previously reported analyses with respect to the significance of results or selected target model (see Tables 4–9 for further details).

#### Prior beliefs and epistemic change

Exploring the relationship between pre-intervention values, instruction (i.e., treatment groups) and latent change scores, we found that treatment effects were descriptively stronger in the more naive group but that these differences failed to reach significance for all outcome measures (all *p* > 0.05).

## Discussion

### Effects of diverging information on epistemic change

#### Hypothesis 1

Surprisingly, confirmatory analyses revealed no significant group differences between experimental groups. Results suggest that this lack of significant findings is largely due to a profound decrease in topic-specific and domain-specific absolutism and multiplism that takes place in our control group. Overall, this trend toward advanced beliefs in the control group and a decrease in multiplism as well as an increase in evaluativism in the “Unresolvable Read” group are the most important deviations from our a priori expected pattern of results concerning Hypothesis 1 (see Table 1). Applying these results to our specific hypotheses H1a, H1b, and H1c, we draw the following conclusions.

##### Hypothesis 1a

The second part of H1a assumed that the learning strategies task in the control group would not induce epistemic change. As stated above, our data clearly point toward a rejection of this hypothesis as advanced beliefs concerning absolutism and multiplism thrive in the control group. How can we explain this unexpected trajectory? After re-inspecting the materials from our control group, we tend to reframe the learning strategies task, i.e., reading texts on students employing different learning strategies, as a presentation of diverging information on the topic of learning strategies. More specifically, participants may interpret each description of a student employing a learning strategy as a “case study” that introduces a new knowledge claim regarding the efficacy of a certain learning strategy. Hence, this presentation of conflicting knowledge claims might engender a decline of absolute beliefs, while the subsequent task that requires participants to compare these knowledge claims on a set of predefined criteria (the adjunct questions) may trigger an integration of diverging information and, therefore, thwart a change toward multiplistic beliefs. Along these lines, selecting the topic “learning strategies” and this kind of control task may have been ill-fated choices with regard to obtaining significant differences between treatment and control groups because both the gender stereotypes interventions and the learning strategies task are settled in the educational psychology domain. Possibly, our subjects perceived learning strategies to be even more prototypical for this domain. Therefore, crossover-effects may exist for beliefs on different topics that are settled within the same domain (i.e., learning strategies and gender stereotyping within educational psychology). On the other hand, these “ill-fated choices” opened up a highly interesting new perspective for examining the diverging information paradigm. Based on our control group, we are actually able to compare effects of the mere presentation of any kind of diverging information, to science-based diverging information that was explicitly designed to evoke epistemic doubt and change toward advanced beliefs.

Nonetheless, as a consequence, the actual effect size of examined effects (and thus the power of our tests) that compared effects of gender stereotype interventions to control groups might be lower than expected for H1a. At least the non-significant effects in confirmatory analyses substantiate this theory. In spite of this fact, exploratory analyses introduce some evidence in favor of H1a as they revealed that topic-specific interventions fostered topic-specific epistemic change toward advanced beliefs when compared to the control group (an increase in the D-Index, a decrease in multiplism and an increase in evaluativism). Interestingly, this finding also holds for psychology-specific justification beliefs (a decreased belief in justification by authority in the “Resolvable Read and Write” and the “Unresolvable Read” group, as well as an increased belief in justification by multiple sources across treatment groups).

In conclusion, H1a can be partially confirmed as we observed some kind of treatment effect on five out of eleven outcome variables. Unexpectedly, the control task induced epistemic change toward advanced beliefs but exploratory analyses revealed that change toward advanced beliefs was more prominent for the treatment groups (in particular, evaluativism did only change in these groups). Additionally, treatment group interventions promoted the development of advanced justification beliefs more efficiently, which indicates that the mere presentation of any kind of diverging information does not equally affect all dimensions of epistemic beliefs.

##### Hypothesis 1b

Contrary to our expectations, changes in evaluativism in the “Unresolvable Read” group were similar to changes in the “Resolvable Read and Write” and “Resolvable Read” groups. Therefore, no significant differences were found for evaluativism between treatment groups. Even more importantly, non-significant effects do not seem to be due to power issues as the “Unresolvable Read” tended to outperform the “Resolvable Read” group—at least on a descriptive level. In a nutshell, our results indicated that epistemic change differed between treatment groups only on one out of eleven outcomes and in this case the observed effect even contradicted the expected pattern of effects (i.e., beneficial effects occurred in the “Unresolvable Read” group). Thus, H1b is completely rejected; the consequences of this will be discussed in the implications section.

##### Hypothesis 1c

The first part of this hypothesis (efficacy in the “Resolvable Read” group) is strongly connected to H1a and, thus, can be regarded as partially confirmed. A precondition for testing the second part of this hypothesis (“difference in effects in the “Resolvable Read” and “Resolvable Read and Write” group is small to moderate”) in a statistically sound way was that the corresponding target model would have been chosen by LRTs. Unfortunately, this was not the case as chosen target models restricted effects to be equal across groups. Therefore, they did not allow to introduce model constraints on effect parameters of dummy-coded intervention groups or to include differences between those effects as additional parameters in our model (i.e., for testing the hypothesis “difference smaller than value x”).

On the other hand, the fact that differences between groups did not become significant based on LRTs implies that overall differences in efficacy cannot be very large because otherwise they would have been detected (as our power analyses indicate). Still, these LRT did not explicitly test the null hypothesis for H1c and descriptive statistics indicate that (small) differences might exist for some outcome measures. In other words, we cannot say for sure if the writing instruction supported epistemic change in our study but we can rule out with some certainty that it was a prerequisite for change. In conclusion, our data tend to confirm the first part of H1c (overall efficacy of the reading task), but are not able to fully test the second part of H1c that pertains to incremental effects of reflecting on diverging information.

#### Hypothesis 2

Our statistical analysis plan prescribed that H2 (i.e., differences in the efficacy concerning domain–and topic-specific measures) was only examined if differences between experimental groups occurred. Due to the fact that no differences between experimental groups (H1) were found in confirmatory analyses, Hypothesis 2 was not tested in our confirmatory analyses.

However, evidence in favor of this hypothesis stems from exploratory analyses, where significantly stronger effects in topic-specific measures were found for the D-Index and for multiplism (when controlling for covariates). Although findings for evaluativism descriptively confirmed this trend, the corresponding effects failed to reach significance. All in all, we found the hypothesized relationship between effects on topic–and domain-specific measures in two out of three cases, in which it could be meaningfully tested, and, therefore, Hypotheses 2 can be regarded as partially confirmed.

Then again, extrapolating from this notion, we would expect to find even weaker differences between effects in our topic-specific intervention groups and our control group for justification beliefs in psychological science, as this is the highest level-domain investigated by our study (i.e., gender stereotypes are a topic within educational psychology, which represents a subdomain of psychological science). Interestingly, this was not the case. On the contrary, we found effects for justification beliefs that would have been significant according to the criteria of our confirmatory analyses. Hence, different dimensions of epistemic beliefs seem to respond in very distinct ways to various aspects of administered interventions. Possibly, the learning strategies control task is only generalized to educational psychology (as a method within this domain), while the resolvable controversies intervention is generalized to both the topic of gender stereotyping and psychological science as a whole (because it deals with research findings on gender stereotypes).

### Implications and further directions

With our first research question, we aimed to create a better understanding of how exactly diverging information affect epistemic change. The findings that we obtained for subjects that received unresolvable controversial information tell a very interesting story in this regard and offer promising starting points for future research. To our surprise, advanced epistemic beliefs (especially justification beliefs) prospered under these circumstances. This is even more remarkable as manipulation check analyses indicated that subjects actually perceived the presented information to be more inconsistent than subjects in the other groups. Why do subjects not regress to simpler multiplistic beliefs when facing this entirely inconsistent information but instead progress to advanced beliefs? Various explanations are conceivable: Possibly, our subjects found some way to integrate conflicting findings and went to great lengths in order to integrate conflicting findings (e.g., by identifying an alternating pattern). Alternatively, they may attribute inconsistencies of presented information solely on the limited amount of information that was offered by our intervention. Especially evaluativists could readily align new information to their existing beliefs by arguing that contextual factors exist but that prior research has, up to now, failed to identify those factors. In accordance with this notion, Rule and Bendixen (2010) argued that schema theory (Anderson et al., 1977) might offer a fruitful framework for understanding the role of prior beliefs in epistemic change. Furthermore, applying our findings to the current situation in psychology (e.g., the replication crisis), one could suggest that ill-structured knowledge does not necessarily hinder individuals' epistemic development after all. Indeed, our results suggest that advanced justification beliefs might prosper under this “climate of contradictoriness.” On the other hand, this also implies that our population's prior competence in integrating conflicting knowledge claims might have been distinctively high. Therefore, it may be questionable if our results can be generalized beyond higher education students in psychology—even though existing research on beneficial effects of “standard” diverging information interventions (Kienhues et al., 2016) possibly corroborates our findings. This body of research also includes quasi-experimental studies from other disciplines whose findings are consistent with our observations in the “Unresolvable Read” group. For example, Han and Jeong (2014) showed that epistemic beliefs of (gifted) high school students who planned to major or majored in science and engineering prospered when they attended a Science-Technology-Society education program. In this education program, they were (among others) confronted with dilemmas in engineering and natural science that—just like the unresolvable controversies in our study—could not be resolved within the course. Nevertheless, these unresolvable dilemmas fostered advanced beliefs and moral judgment (Han and Jeong, 2014). As a consequence, future research should examine, which degree of inconsistency fosters epistemic development and from when on it hinders progress, while paying close attention to the role of prior beliefs and educational background. Conceptual change research on “dissonance producing approaches” (e.g., contrasting common misconceptions to scientists' views) for teaching and their limitations (c.f. Clement, 2013) should provide some valuable input for this purpose.

Concerning our second research question, which aimed at investigating effects of reflecting on diverging information, results are harder to interpret. However, the concept of “epistemic reflexivity” that was introduced by Feucht et al. (2017) as an internal dialog that is focused on “personal epistemologies leading to action for transformative practices in the classroom” (p. 234) might be able to shed some light on the observed pattern of effects. The effects of *reflection* may not be very large because reflecting on diverging information lacks goal-orientation (i.e., the goal of epistemic change was not explicitly given in the writing task instructions). Hence, Lunn Brownlee et al.'s (2017) framework for epistemic reflexivity might be applied when designing future epistemic change interventions in order to ensure that reflection leads to reflexive thinking. Framing the same argument in Bendixen and Rule's model (Bendixen and Rule, 2004; Rule and Bendixen, 2010), one could also reason that subjects' “will” to resolve epistemic doubt (i.e., epistemic volition) may have been insufficient. Since epistemic doubt, epistemic volition and resolution strategies are thought to be part of higher order mechanisms in their model (Rule and Bendixen, 2010), larger effects of reflecting on diverging information might become apparent if subjects' epistemic volition is simultaneously targeted by interventions. Therefore, even though this is somewhat speculative, our results could point to the importance of epistemic volition in epistemic change, an aspect that should be investigated in future research. One way to do so would be the design of intervention components that are tailored specifically to affect *epistemic doubt, epistemic volition* or *reflection* and to investigate their incremental effects on epistemic change.

Moreover, our study gave some interesting insights into how effects of topic-specific interventions are generalized—a pressing issue in epistemic change research (cf. Bråten, 2016). In fact, experimental studies often possess a narrow topic-specific scope (cf. Muis et al., 2016) and, therefore, their overall impact on an individual's more general epistemic development may be questionable (cf. Bråten, 2016). With regard to this concern, Kienhues et al. (2008) have argued that topic-related epistemic cognitions can be used to exemplify notions beyond this topic. Thus, their so-called *exemplary principle* predicts that a certain way of dealing with epistemic problems can be transferred when approaching problems in related areas. Our research corroborates to this notion. As could have been predicted by the *exemplary principle*, we found carry over effects within the domain of educational psychology: Topic-specific intervention effects of our gender stereotyping intervention were transferred to domain-specific beliefs and even to higher-level justification beliefs.

Furthermore, the presentation of diverging information on the topic of learning strategies caused an unexpected decrease in absolute beliefs regarding another topic within the same domain (i.e., gender stereotyping within educational psychology). However, not all topic-specific beliefs were equally affected. More specifically, diverging information on learning strategies did not result in significant changes in evaluativism (topic–or domain-specific) nor in justification beliefs. This yields two important implications which pertain to both our first and last research question: First, the generalization of epistemic beliefs seems to depend on the dimension of epistemic beliefs under investigation. Possibly, it is comparatively easy to change beliefs on the structure of knowledge (i.e., certainty and simplicity) by presenting (any kind of) diverging information that is settled within a certain domain. In contrast, changing other belief dimensions (e.g., justification beliefs) might require interventions that are specifically tailored to modify epistemic beliefs. Future research should address this question, where Greene et al. (2008, 2010) distinction between ontological beliefs and epistemic beliefs may prove to be a valuable starting point for this endeavor. Secondly, we saw that evoking doubt regarding absolute beliefs was comparatively easy as we required no didactical concept in order to change those beliefs. Our learning strategies task efficiently reduced topic–and domain-specific absolute beliefs—at least in the short term—even though it was actually designed as a control task. Drawing upon this thought, epistemic change interventions that aim at a simple reduction of absolutism might lack in ambition because individuals are likely to encounter a vast amount of diverging information in their everyday life (in particular in softer disciplines and/or in higher education). Additionally, our findings suggest that these insights might be readily conferred to adjacent domains. However, once more, specific characteristics of our sample have to be taken into account when interpreting these findings and future research should examine if our observed pattern of effects holds in confirmatory studies for other populations.

### Limitations

First, one may criticize that findings and conclusions of our study are largely based on exploratory analyses. However, our exploratory analyses modified confirmatory analyses in no substantial way as we derived exploratory analyses and outcomes from our prespecified theory and did not alter our research questions or hypotheses. Instead, we investigated the same questions on a more basic level in order to meaningfully examine if the overall paradigm had worked as intended. Nonetheless, as for all exploratory research, it is the task of future confirmatory studies to validate our findings. Until then, these findings should be cautiously interpreted.

Secondly, the duration of our intervention was rather short. This is particularly true considering the mismatch between intervention duration and length of normative development process that the intervention aims at. However, this is not uncommon for this kind of intervention (cf. Muis et al., 2016) and is indeed well-founded, as this experimental setting allows to disentangle the mechanism of change in the first place. Moreover, to settle the issue of targeting a long-term process by short-term interventions, Ferguson et al. (2012) referred to Vygotsky (1978). Based on his framework, they argued that short-term interventions in an experimental setting might be able to accelerate or compress development processes that normally require longer periods of time. Nonetheless, long-term effects of those short-term interventions should be investigated in future studies by including follow-up measurements.

Concerning the power of our analyses, the significance criteria might have been chosen too restrictive for some exploratory analyses. We used the standard *p* < 0.05 criteria for likelihood ratio tests although we wanted to inspect one-sided effects in some cases. This procedure was designed to avoid an increased Type I error rate because of multiple testing when comparing effects for multiple treatment groups simultaneously. Unfortunately, the power in the equal group effects model of our exploratory analyses may have been diminished because only one intervention effect is estimated within this model and, thus, multiple testing is not an issue here. As a consequence, in some analyses, we obtained no significant LRT while the (single) parameter estimate would have been significant according to our criteria. Ceiling effects may further contribute to these power issues. However, exploratory analyses revealed that the intervention efficacy did not vary depending on the developmental level of epistemic beliefs. This possibly indicates that all groups were equally affected by ceiling effects (if at all). On the other hand, the existence of those ceiling effects further justifies our choice of the D-Index as exploratory outcome which does not suffer from this issue.

### Conclusion

In sum, this study illustrates that many questions remain unanswered when it comes to understanding the relationship between (properties of) diverging information, epistemic doubt and subsequent changes on different dimensions of epistemic beliefs. It shows that evoking doubt regarding absolute beliefs is relatively easy because individuals seem to be skillful in recognizing varying knowledge claims and subsequently averting absolute beliefs. Additionally, we found evidence for the existence of carry-over effects from topic-specific interventions for both higher-level domain-specific beliefs (i.e., beliefs regarding educational psychology and psychological science as a whole) and beliefs pertaining to other topics within the same domain (i.e., effects of the learning strategies task on beliefs on gender stereotyping). In this context and for epistemic change in general, the role of reflecting on presented conflicting information should be thoroughly addressed by future research. Finally, we may need to reconsider our understanding on how individuals acquire and retain evaluativistic beliefs and the role that non-resolvable controversial information play in this development.

## Data availability statement

The data generated and analyzed for this study can be found in PsychArchives: doi: 10.23668/psycharchives.930.

## Author contributions

TR and MK conceived, planned and preregistered the experiment based on a project proposal written by TR. MK conducted the study, analyzed the data and prepared the first draft of the manuscript. TR reviewed critically, revised the article and supervised the project.

### Conflict of interest statement

The authors declare that the research was conducted in the absence of any commercial or financial relationships that could be construed as a potential conflict of interest.

## Footnotes

^1^In the following, we will refer to all experimental groups that received any kind of diverging information on gender stereotyping as *treatment groups* or *treatment conditions* (i.e., irrespectively of the (un)resolvable nature of these information or if subjects had to write an integrating text on these controversies).

^2^This is a minor modification to the planned procedure in our preregistration which suggested using the “Resolvable Read and Write” group as reference. However, this modification does not substantially affect our confirmatory analyses as it only changes how the model is parameterized (and not if effects become significant or not). We chose this procedure as it allowed us a more convenient interpretation of results (i.e., in terms of consistency with exploratory analyses).
